# Salvianolic acid B inhibits the proliferation and metastasis of A549 lung cancer cells via miR-23a/PTEN/AKT pathway

**DOI:** 10.1038/s41598-025-32336-9

**Published:** 2025-12-22

**Authors:** Ye Yang, Lei Huang, Li Dai, Xin Zhou, Bingjun Qian

**Affiliations:** 1 Department of Chinese Traditional Medicine, Jiangsu Medical College, Yancheng, 224005 Jiangsu P.R. China; 2 Department of Basic Medical Sciences, Jiangsu Medical College, Yancheng, 224005 Jiangsu P.R. China; 3 Department of Preventive Medicine, Jiangsu Medical College, 283 South Jiefang Road, Yancheng, 224005 Jiangsu P.R. China

**Keywords:** Salvianolic acid b, A549, miR-23a-3p, PTEN, Proliferation, Metastasis, Cancer, Cell biology, Chemical biology

## Abstract

Salvianolic acid B (Sal B), a polyphenolic compound with potential anti-cancer properties, possesses mechanisms of action that remain incompletely understood. This study aimed to elucidate the effects of Sal B on the A549 cell line and to investigate the underlying molecular mechanisms. In this study, A549 cells were co-cultured with varying concentrations of Sal B for 48 h. We utilized MTT, wound healing, and transwell assays to evaluate cell proliferation, migration, and invasion, respectively. The expression levels of miR-23a-3p, E-cadherin, N-cadherin, Snail, PTEN, and p-AKT were analyzed using western blotting and RT-PCR. Additionally, a xenograft model of transplanted A549 tumors was employed to assess the in *vivo* antitumor effects of Sal B. Immunohistochemistry (IHC) was utilized to measure the expression of cleaved PARP, E-cadherin, N-cadherin and Snail. Our findings demonstrated that Sal B inhibited A549 cell proliferation, migration, and invasion in a dose-dependent manner. Furthermore, in *vivo* experiments revealed that Sal B significantly suppressed the growth and metastasis of A549 tumors, as evidenced by elevated levels of cleaved PARP and E-cadherin, along with a marked reduction in N-cadherin and Snail compared with the control group. Importantly, we observed that Sal B significantly downregulated miR-23a-3p expression, whereas the overexpression of miR-23a-3p via transfection attenuated the inhibitory effects of Sal B on A549 cell proliferation and metastasis. Western blot results indicated that the downregulation of miR-23a-3p led to the upregulation of PTEN and the downregulation of p-AKT. Collectively, these findings suggest that Sal B’s mechanism of action in NSCLC may involve the modulation of the miR-23a/PTEN/AKT signaling pathway, thereby highlighting its potential as a therapeutic candidate for NSCLC.

**Supplementary Information:**

The online version contains supplementary material available at 10.1038/s41598-025-32336-9.

## Introduction

Non-small cell lung cancer (NSCLC) is the most prevalent subtype of lung cancer, accounting for approximately 85% of cases^1^. Despite recent advances in chemotherapy and targeted therapies that have improved survival rates, the survival prognosis for patients with metastatic NSCLC remains poor^2^. Additionally, the emergence of drug resistance and toxic side effects associated with conventional treatments has resulted in diminished treatment efficacy^3,4^. In this context, the screening of anticancer active compounds derived from natural resources is crucial for enhancing NSCLC treatment and has emerged as a primary focus of scientific research.

*Salvia miltiorrhiza Bunge*, commonly known as Danshen, is widely acknowledged for its efficacy in promoting blood circulation and alleviating blood stasis^5^. Furthermore, Danshen has been employed in the treatment of other ailments, including cancer^6^. Salvianolic acid B (Sal B), a principal water-soluble polyphenolic compound derived from Danshen roots, has exhibited therapeutic effects against various cancers, including NSCLC, breast cancer, head and neck squamous cell carcinoma, gastric cancer, and colorectal cancer^7^. Notably, Li et al. (2014) reported that Sal B inhibited the growth of NSCLC A549 cells, with an IC_50_ value of 279.6 µM^8^. Furthermore, recent studies have demonstrated that Sal B effectively inhibits TGF-β1-induced epithelial-mesenchymal transition (EMT) and migration in A549 cells, which are critical processes in the metastasis of NSCLC^9^. Based on these findings, it can be posited that Sal B may have potential as a therapeutic agent for NSCLC. However, further investigation is required to elucidate the precise molecular mechanisms by which Sal B inhibits the proliferation and metastasis of NSCLC.

MicroRNAs (miRNAs) are endogenous small non-coding RNAs of 18–22 nucleotides that play an essential role in regulating various biological processes, including cell proliferation, metastasis, apoptosis, and metabolism^10–12^. It has been established that they regulate gene expression through base pairing with the 3′-untranslated region (3′-UTR) of target mRNAs. Previous research has indicated that aberrant miRNAs are involved in the growth and metastasis of NSCLC^13^. For instance, miR-21 is frequently overexpressed in NSCLC, thereby promoting tumor growth and metastasis by targeting tumor suppressor genes, including PTEN and PDCD4^14^. Additionally, the level of miR-155 is also up-regulated in NSCLC, where it facilitates cancer cell proliferation and invasion by activating the PI3K/AKT signaling pathway^15^. These findings reveal a critical mechanism through which miRNAs regulate the growth and metastasis of NSCLC. Consequently, further investigation into the function and role of miRNAs in NSCLC is imperative.

In this study, we aimed to examine the effects of Sal B on NSCLC and to elucidate the underlying mechanisms. The inhibitory effects of Sal B treatment on the growth and metastasis of A549 cells were analyzed in vitro and in vivo. Furthermore, the expression levels of PTEN, p-AKT, and E-cadherin were evaluated. Further investigations revealed that Sal B significantly down-regulated the expression of miR-23a and inhibited the PTEN/PI3K/AKT signaling pathway, suggesting its potential as a monotherapy or in combination therapy for NSCLC.

## Materials and methods

### Materials and chemicals

Salvianolic acid B (Sal B) used in this research was purchased from Nantong FeiYu Biotechnology Co., Ltd. (purity > 98%). MTT (3-(4,5-dimethylthiazol-2-yl)-2,5-diphenyltetrazolium bromide), dimethyl sulfoxide (DMSO), and methylene blue were obtained from Beyotime Co., Ltd. (Nanjing, China). McCoy’s 5 A medium, fetal bovine serum (FBS), L-glutamine, penicillin, streptomycin, and trypsin were acquired from Keygen BioTECH Co., Ltd. (Nanjing, China). Balb/c nude mice (5–7 weeks old) were provided by Beijing Vital River Laboratory Animal Technology Co., Ltd. (Beijing, China). All other chemicals were of reagent-grade quality. A 10 mM stock solution of Sal B was prepared in DMSO and stored at − 20 °C, protected from light.

### Cell culture

All cell lines utilized in this study were procured from the National Biomedical Laboratory Cell Repository in Shanghai, China. The human A549 cancer cell line was cultivated in McCoy’s 5 A medium (Invitrogen; Thermo Fisher Scientific, Inc.), with the supplement of 10% (v/v) fetal bovine serum (FBS; Sigma-Aldrich; Merck KGaA) and 1% penicillin-streptomycin (Sigma-Aldrich; Merck KGaA). The cells were maintained at 37 °C in a humidified incubator with 5% CO_2_.

### MTT assays

A549 cells were seeded in 96-well plates at a density of 2 × 10³ cells per well. The cells were exposed to Sal B at concentrations of 0, 25, 50, and 100 µM for various durations. Following treatment, cells were harvested at predetermined time points and incubated with 20 µL of a 5 mg/mL MTT solution for 2 h. Thereafter, the supernatant was subsequently removed, and the cell pellet was resuspended in 150 µL of dimethyl sulfoxide (DMSO) that had been added to each well. The plate was subjected to agitation at 37 °C for 20 min to dissolve the formazan crystals. Subsequently, the absorbance values were then measured at a wavelength of 490 nm. The cell growth inhibition rate was calculated using the following formula: cell growth inhibition rate = (1 - values of each sample / value of control) × 100%.

### Colony formation assays

A549 cells were seeded at a density of 600 cells per well and exposed to various concentrations of Sal B for 48 h. Following this exposure, the cells were permitted to proliferate in fresh culture medium for 14 d. Subsequent to the incubation period, the cells were fixed with 4% paraformaldehyde (Beyotime, China) for a minimum of 10 min and subsequently washed with distilled water for 3 min. The cells were then stained with Crystal Violet Staining Solution (Beyotime, China) for 10 min. This was followed by thorough washing with distilled water prior to observation and imaging. The quantification of colonies was conducted using the image processing and analysis in Java software (ImageJ v1.43f, https://imagej.net/software/imagej/).

### Wound-healing assays

A wound-healing assay was conducted to evaluate the migratory potential of A549 cells. The cells were seeded in 6-well plates at a density of 3 × 10^5^ cells per well and incubated until they reached 90% confluence. A wound was then created in the cell monolayer by scraping it with a 200-µL pipette tip, followed by washing with phosphate-buffered saline (PBS) to remove detached cells. The cells were subjected to an incubation for 48 h in McCoy’s 5A medium, which had been supplemented with 8% fetal bovine serum (FBS), and at varying concentrations of Sal B. After the incubation, the medium was replaced with PBS. The wound gap was imaged using an Olympus BX53 microscope equipped with a digital camera (Olympus, Tokyo, Japan).

### Transwell assays

To prepare the matrix-coated surface, the upper side of the transwell membrane was coated with 6% matrix gel and then incubated at 37 °C in a CO_2_-humidified incubator for 2 h. Following the dosing cycle, A549 cells were harvested, and the culture medium was removed by a centrifugation at 1000 rpm for 4 min. The cells were then washed twice with phosphate-buffered saline (PBS) and then resuspended in serum-free medium. Subsequently, 5 × 10^5^ cells were added to the Transwell chamber and incubated at 37 °C in a CO_2_-humidified incubator for 48 h. Following this, the Transwell chamber was removed, and the culture medium was discarded. The cells were washed twice with calcium-free PBS, and non-migrated cells were gently wiped away using a cotton swab. The cells were fixed with 4% formaldehyde, stained with 1% crystal violet for 15 min, and washed three times with PBS. Residual moisture was then gently removed, and then the cells were imaged using an upright biological microscope (Olympus BX53, Tokyo, Japan).

### RNA extraction and quantitative real-time PCR

Total RNAs were isolated from A549 cells treated with Sal B (100 µM) using Trizol^®^ (Thermo Fisher Scientific, Inc.) according to the manufacturer’s instructions. Subsequently, quantitative real-time polymerase chain reaction (qRT‐PCR) was then performed on a Bio‐Rad CFX‐96 system (Bio‐Rad) by using the following primers (Table 1). The PCR reactions were replicated at least three times, and the Ct values were used to analyze the relative expression levels of different genes.

**Table 1:** Primer sequences for reverse transcription and real-time RT-PCR.

Primer	RT	Forward	Reverse
General RT primer	5'-GTCGTATCCAGTGCAGGGTCCGAGGTATTCGCACTGGATACGAC’3'		
General Reverse primer			5′-AGTGCAGGGTCCGAGGTATT‐3′
miR-1297	5′-CACCTG‐3′	5′-CGCGCGCGTTCAAGTAATT‐3′	
miR-5011-5p	5′-GAGTGC‐3′	5′-CGCGCGTATATATACAGCCAT‐3′	
miR-23a-3p	5′-GGAAAA-3′	5′-ATCACATTGCCAGGGATT-3′	
miR-5692c	5′-GTACAC‐3′	5′-CGCGCGAATAATATCACAGTAG‐3′	
miR-26a-5p	5′-AGCCTA‐3′	5′-CGCGTTCAAGTAATCCAGGA‐3′	
miR-26b-5p	5′-ACCTAT‐3′	5′-GCGCGTTCAAGTAATTCAGG‐3′	
miR-23c	5′-GGGTAA‐3′	5′-CGCGATCACATTGCCAGTGA‐3′	
miR-23b-3p	5′-GTGGTA‐3′	5′-CGATCACATTGCCAGGGAT‐3′	
miR-4465	5′-TCCCCT‐3′	5′-CGCGCTCAAGTAGTCTGACC‐3′	
miR-5692b	5′-ACACCT‐3′	5′-GCGCGCGAATAATATCACAGT‐3′	

### Adenovirus transfection experiments

The effect of miR-23a-3p overexpression on cells was investigated by means of Adenovirus transfection experiments. A549 cells in the exponential growth phase were plated onto 6-well plates in a growth medium devoid of antibiotics for 24 h. Subsequently, adenoviruses harboring miR-23a-3p mimic (AD-miR-23a-3p) and its non-specific control (AD-NC) (Genechem, Shanghai, China) were transfected following the manufacturer’s instructions. These were utilized in subsequent experiments.

### In vivo tumor xenograft experiments

Six-week-old female Balb/c nude mice (weighing 18 ± 2 g) were obtained from Qinglongshan Animal Breeding Farm (Nanjing, Jiangsu). The xenograft model of A549 cells in nude mice was established according to the method reported by Huang L et al.^16^. A549 cells (5 × 10^6^ cells/mL) were subcutaneously injected into the right flank of each mouse. After 7 days of incubation, 15 mice with tumor volumes ranging from 90 to 100 mm³ were randomly assigned to three groups (*n* = 5/group): a control group, a High-dose Sal B group (100 mg/kg Sal B), and a low-dose Sal B group (50 mg/kg Sal B). The solutions were administered via intraperitoneal injection (i.p.). The control group received an equivalent volume of physiological saline. Tumor volumes were calculated using the formula: [length × (width²)]/2. At the end of the study, animals were euthanized by cervical dislocation, and tumors were collected for further analysis. An endpoint was established at a tumor volume exceeding 1500 mm³ or body weight loss exceeding 25%. At the humane and experimental endpoints, mice were euthanized by cervical dislocation, with death confirmed by the cessation of respiratory and cardiac function. All mice were housed under specific pathogen-free (SPF) conditions, according to the Guidelines and Regulations for the Use and Care of Animals set forth by the Center for Laboratory Animal Care of Jiangsu Medical College. The mice were maintained in a pathogen-free environment with a temperature of 23 ± 2 °C, humidity of 55 ± 5%, and a light cycle of 11 h light and 13 h dark. The mice had free access to food and water throughout the study. The health and weight of the mice were monitored at three- to four-day intervals. All animal procedures received approval from the Ethics Committee of Jiangsu Medical College and were conducted in accordance with institutional guidelines and regulations to minimize animal suffering.

### Western blotting

The Western blot analysis was performed according to previously described methods^17^. A549 cells were cultured in McCoy’s 5 A medium supplemented with 8% fetal bovine serum (FBS) at 37 °C in a humidified incubator with 5% (v/v) carbon dioxide (CO_2_). During the logarithmic growth phase, cells were seeded at a density of 1 × 10^7^ cells per dish in 60 mm × 15 mm dishes to allow for overnight adherence. Following 72 h of treatment with Sal B and dimethyl sulfoxide (DMSO), total protein was extracted from the treated A549 cells or tumor tissue using 1 mL of RIPA cell lysis buffer (Solarbio, R0010), which was supplemented with 10 µL of phenylmethylsulfonyl fluoride (Beyotime, ST506), 10 µL of protease inhibitor (Solarbio, P0100), and 10 µL of phosphatase inhibitor (Solarbio, P1260). The protein concentration was determined using a BCA protein assay kit (Beyotime, P0010S) following the lysis the total cell solution on ice for 1 h. The proteins were then subjected to heating at 100 °C for 10 min and subsquently stored at -20 °C. For Western blotting assays, proteins were loaded onto SDS-PAGE gels and transferred onto a polyvinylidene difluoride (PVDF) membrane (Millipore, Billerica, USA). After the protein transfer, the membranes were blocked with 5% (w/v) skimmed milk and then incubated with primary antibodies against PTEN (CST, USA), phosphorylated AKT (p-AKT) (CST, USA), E-cadherin (CST, USA), N-cadherin (CST, USA) and Snail (CST, USA) for 12 h at 4 °C, followed by incubation with HRP-conjugated secondary antibodies (1:2000) for 1 h at room temperature for detection. The expression levels of the proteins were then normalized to β-tubulin, and the protein bands were quantified and analyzed using ImageJ software.

### Immunohistochemistry assays

Tumor samples were collected on day 18 from Balb/c mice bearing A549 tumors. The tumors were fixed in 4% paraformaldehyde for 48 h and subsequently sectioned longitudinally. The samples were then embedded in paraffin, treated with 0.3% hydrogen peroxide for 60 min, and blocked with 1% BSA. Sections were incubated overnight at 4 °C with primary antibodies, followed by a 60-minute incubation with secondary antibodies at room temperature. Each group of sections was examined using a DM6B fluorescence microscope, and images were randomly acquired from each slide. Semi-quantitative image analysis was conducted to measure the average optical density (AOD) of cleaved PARP, E-cadherin, N-cadherin and Snail expression using ImageJ software.

### Statistical analysis

The results are presented as the mean ± standard deviation (SD) derived from a minimum of three independent experiments. Statistical analyses were conducted using SPSS software (version 19.0). Group comparisons were performed using either the student’s t-test or one-way ANOVA, as appropriate. A p-value of less than 0.05 was considered to be statistically significant.

### Ethics approval and consent to participate

Experimental animals were handled under a protocol approved by the Center for Laboratory Animal Care, Jiangsu Experimental Animal Ethics Committee, and Natural Science Foundation of the Jiangsu Higher Education Institutions of China (21KJB360013).

## Results

### Sal B significantly reduced cell viability of A549 cells

To investigate the inhibitory effect of Sal B on the proliferation of A549 cells, cell viability was assessed at these time points to evaluate the effects of treatment with serial drug concentrations. As shown in Table 2, the results indicated that treatment with Sal B at concentrations of 50 µM or higher significantly inhibited cell growth. Notably, a concentration of 100 µM Sal B induced approximately 68.23% inhibition of proliferation (*P* < 0.01). The highest rate of cell death occurred after 48 h of treatment, with an IC_50_ value of 70.2 µM. Consequently, concentrations of 50 µM and 100 µM were utilized in subsequent experiments. The findings indicate that the antiproliferative effect of Sal B on A549 lung cancer cells is both concentration- and time-dependent.

**Table 2:** Inhibition rate of Sal B on A549 cells at different concentrations and time ($\:\stackrel{-}{x}\pm\:\mathrm{s})$.

Time	Inhibition rate (%)				
	0 µM	25 µM	50 µM	100 µM	
0 h	0.02 ± 0.01	0.05 ± 0.02	0.07 ± 0.02	0.13 ± 0.05	
12 h	0.04 ± 0.02	12.35 ± 2.38^**^	21.35 ± 2.68^**^	39.65 ± 4.23^**^	
24 h	0.07 ± 0.04	19.56 ± 2.65^**^	32.56 ± 5.32^**^	45.62 ± 4.25^**^	
48 h	0.13 ± 0.06	29.16 ± 5.23^**^	44.23 ± 6.23^**^	68.23 ± 7.65^**^	

A549 cells were treated with Sal B at various concentrations: 0, 25, 50 and 100 µM, respectively. A549 cells were counted at various time points (0, 12, 24 and 48 h) after treatment, and inhibition rate (%) was expressed as mean ± SD. **P < 0.01 vs. control.

### Sal B inhibited the migration and invasion of A549 cells

**Fig. 1:**
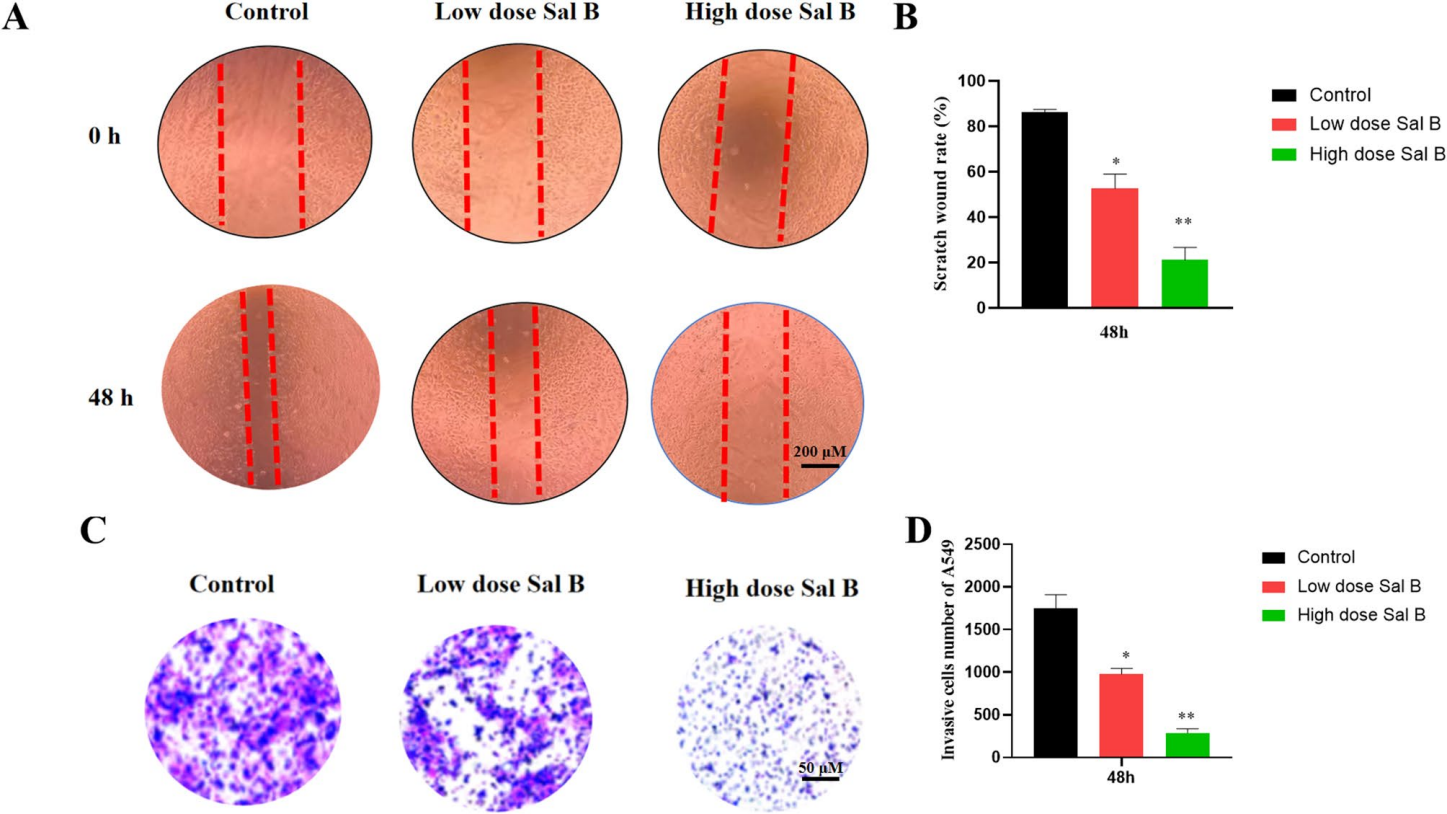
Sal B inhibits the metastatic ability of tumor cells in *vitro.* (**A**) Wound healing assay demonstrated that Sal B resulted in a slower closing of scratch wounds. (**B**) The scratch closure was recorded at 48 h. Scratch wound rate = (0 h scratch width- 48 h scratch width)/0 h scratch width×100%. (**C**) The results of the transwell invasion assay were measured as the number of invaded cells. (**D**) The quantifications of cell invasion are presented as invading cell numbers. Data were analyzed using one-way ANOVA followed by Tukey’s multiple-comparisons test with data shown as mean ± SD; *n* = 3. Scale bar, 50–200 μm. * Indicated the statistical significance between control group and low / high dose Sal B group. *, *P* < 0.05, **, *P* < 0.01.

The effects of Sal B on the migration and invasion of A549 cells were evaluated using wound healing and transwell assays. As illustrated in Figs. 1A /B, the wound healing assay revealed that Sal B significantly inhibited cell migration across various concentrations. The closure of A549 cell wounds was notably delayed with increasing doses of Sal B. Furthermore, the transwell assays demonstrated that Sal B markedly reduced the invasive capacity of lung cancer cells at both high and low concentrations in comparison to the control group (Figs. 1C/D). These findings exhibited the anti-metastatic potential of Sal B.

### Sal B inhibits metastasis of A549 cells by upregulating PTEN

**Fig. 2:**
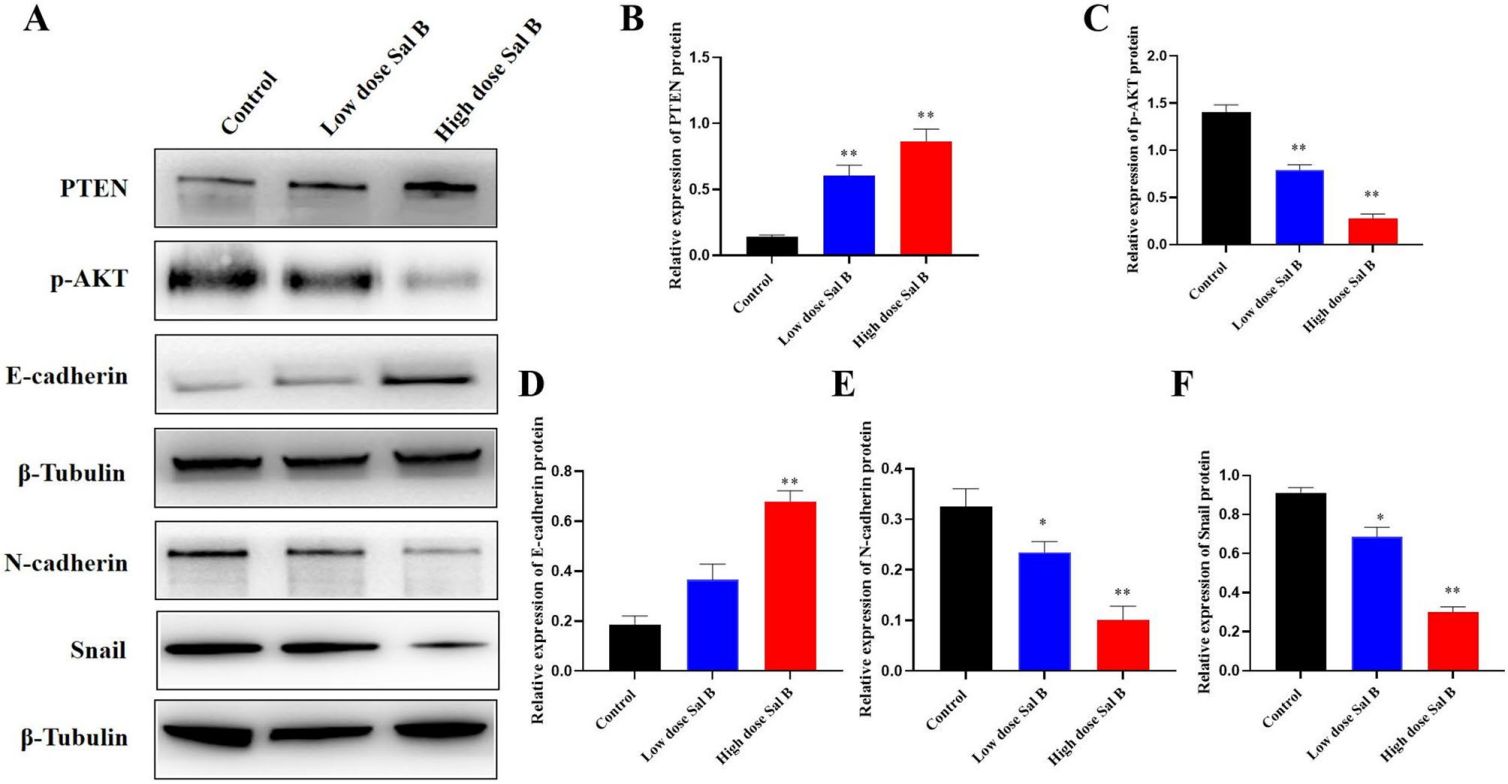
Effect of Sal B on A549 tumor metastasis and the PTEN signaling pathway in *vitro*. (**A**) Western-blotting analysis of the levels of PTEN, p-AKT, E-cadherin, N-cadherin and Snail in A549 cells. Bands were cropped from different parts of the same gel. (**B**) ImageJ analyzed the effects of Sal B on the expression of PTEN. (**C**) ImageJ analyzed the effects of Sal B on the expression of p-AKT. (**D**) ImageJ analyzed the effects of Sal B on the expression of E-cadherin. (E) ImageJ analyzed the effects of Sal B on the expression of N-cadherin. (**F**) ImageJ analyzed the effects of Sal B on the expression of Snail. Original blots/gels are presented in Supplementary Figure S2. Results from independent experiments were statistically analyzed using one way ANOVA: **P < 0.01 compared with control.

The PTEN/PI3K/AKT signaling pathway is integral to various cellular processes, including proliferation, apoptosis, and metastasis. PTEN is a crucial tumor suppressor, essential for maintaining normal cellular functions and preventing tumor initiation and metastasis by modulating the PI3K/AKT signaling pathway. This study aimed to investigate the relationship between the inhibitory effect of Sal B on A549 tumor metastasis and the PTEN signaling pathway. As illustrated in Fig. 2, the present findings demonstrate that Sal B upregulates PTEN expression and downregulates phospho-AKT (p-AKT) in a dose-dependent manner. Furthermore, treatment with Sal B increased the protein level of E-cadherin, whilst concomitantly reducing levels of Snail and N-cadherin, in A549 cells, in a dose-dependent manner (Fig. 2). Collectively, these results suggest that Sal B inhibits A549 tumor cell metastasis through the PTEN/PI3K/AKT signaling pathway.

### Sal B suppressed the proliferation and metastasis of A549 cells by downregulating miR-23a-3p

**Fig. 3:**
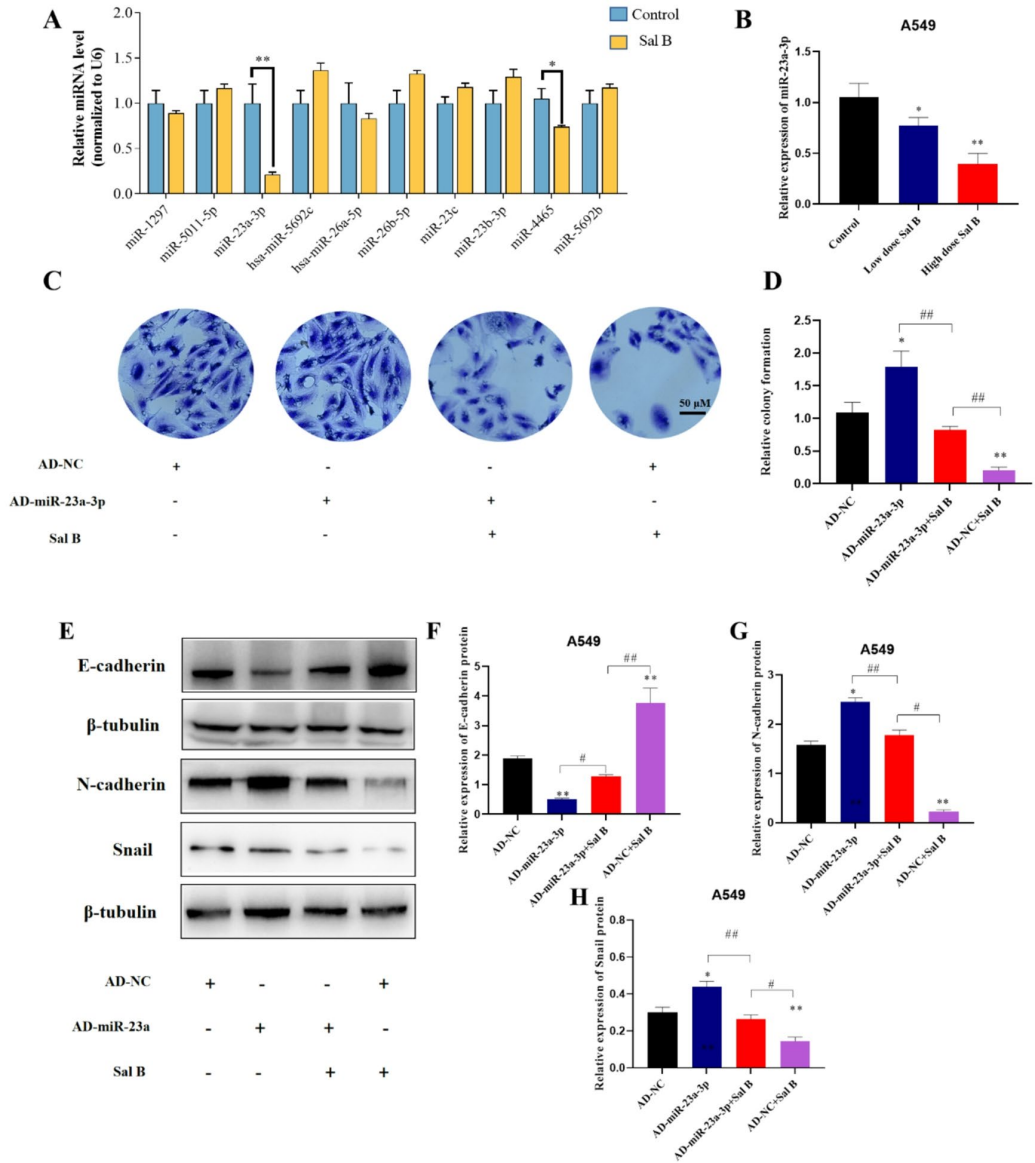
Sal B suppressed the proliferation and metastasis of A549 cells by downregulating miR-26a-3p. (A)The A549 cells were treated with DMSO (0.5%) or Sal B (100 µM) for 24 h, and RT-PCR detected the miRNA expression. (B) RT-PCR measured the miR-23a-3p expression. (C) A549 cells was transfected with equal doses of adenovirus with miR-NC (AD-NC) or miR-23a mimic (AD-miR-23a) for 24 h before exposure of the A549 cells to Sal B. Then, A549 cells were exposed to the concentration of Sal B and colony formation experiment was detected after 48 h. (D) The quantifications of colony formation are presented as invading cell numbers. (E) E-cadherin, N-cadherin and Snail protein level was analyzed by Western blot. The grouping of gels/blots were cropped from different parts of the same gels. (F) Quantification of E-cadherin proteins by Image J. (G) Quantification of N-cadherin proteins by Image J. (H) Quantification of Snail proteins by Image J. Original blots/gels are presented in Supplementary Figure S3. Statistical analysis was performed by one-way ANOVA. *P < 0.05, **P < 0.01 compared with the AD-NC. #*P* < 0.05, ##*P* < 0.01 compared with the AD-miR-23a + Sal B.

In our previous study, a novel mechanism was identified by which Sal B suppresses the proliferation and metastasis of A549 cells via PTEN upregulation. In consideration of the post-transcriptional regulation of gene expression by miRNAs, the miRDB tool was used to predict potential miRNAs that may target the 3’-UTR of PTEN, resulting in the identification of ten candidates (target score ≥ 99). Of these, miR-1297, miR-23a-3p, miR26a-5p, and miR-4465 were downregulated in Sal B-treated A549 cells, with miR-23a-3p exhibiting the greatest reduction (Fig. 3A). The subsequent investigation focused on ascertaining whether Sal B modulates the expression of miR-23a-3p in A549 cells. RT-PCR analysis demonstrated dose-dependent downregulation of miR-23a-3p by Sal B (Fig. 3B). Furthermore, the effect of Sal B on proliferation and metastasis induced by miR-23a-3p overexpression was evaluated. The miR-NC (AD-NC) or miR-23a mimic (AD- miR-23a) was applied to transfect A549 cells. RT-PCR analysis result revealed a 3.8-fold increase in the expression of miR-23a-3p following transfection (see the supplementary information, Fig. S1). The results showed that the overexpression of miR-23a-3p significantly enhanced the proliferation of A549 cells and promoted epithelial-mesenchymal transition (EMT), as evidenced by the decreased expression of E-cadherin and increased expression of N-cadherin and Snail (Fig. 3C-H). However, the treatment of Sal B reversed these effects, thereby indicating that Sal B inhibits A549 cell migration by modulating the expression levels of miR-23a-3p and upregulating the expression levels of PTEN.

### Sal B mediated the miR-23a targets PTEN

**Fig. 4:**
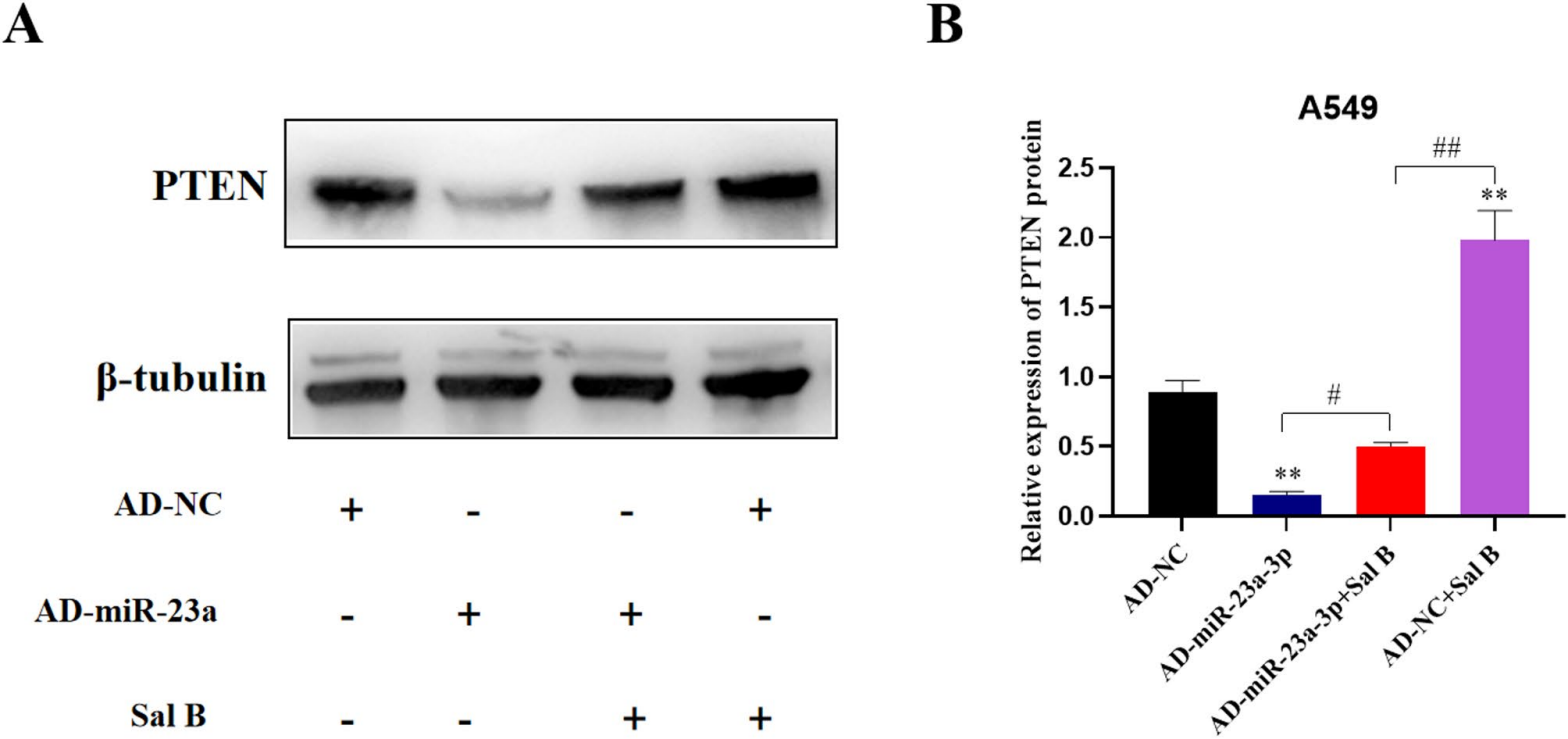
Effect of Sal B on PTEN protein. (A) A549 cells was transfected with equal doses of adenovirus with miR-NC (AD-NC) or miR-23a-3p mimic (AD-miR-23a-3p) for 24 h before exposure of the A549 cells to Sal B. Then, A549 cells were treated with Sal B, and PTEN protein expression was assessed using western blot analysis 48 h later. The grouping of gels/blots were cropped from different parts of the same gels. (B) Quantification of PTEN proteins by Image J. Original blots/gels are presented in Supplementary Figure S4. Statistical analysis was performed by one-way ANOVA. **P < 0.01, compared with the AD-NC. #*P* < 0.05, ##*P* < 0.01 compared with the AD-miR-23a + Sal B.

Following the identification of PTEN as a target of miR-23a-3p, the regulatory role of miR-23a-3p on PTEN expression was investigated. Therefore, the A549 cells were subjected to transfection using either either a negative control (AD-NC) or a mimic of miR-23a-3p (AD-miR-23a-3p). As illustrated in Fig. 4, treatment with Sal B resulted in an increase in PTEN expression. However, the overexpression of miR-23a-3p reversed this effect, leading to a reduction in PTEN protein levels. The findings indicated a possible mechanism through which Sal B enhances PTEN protein expression by decreasing the level of miR-23a-3p.

### Sal B suppressed tumor growth on A549 tumor xenograft mice

**Fig. 5:**
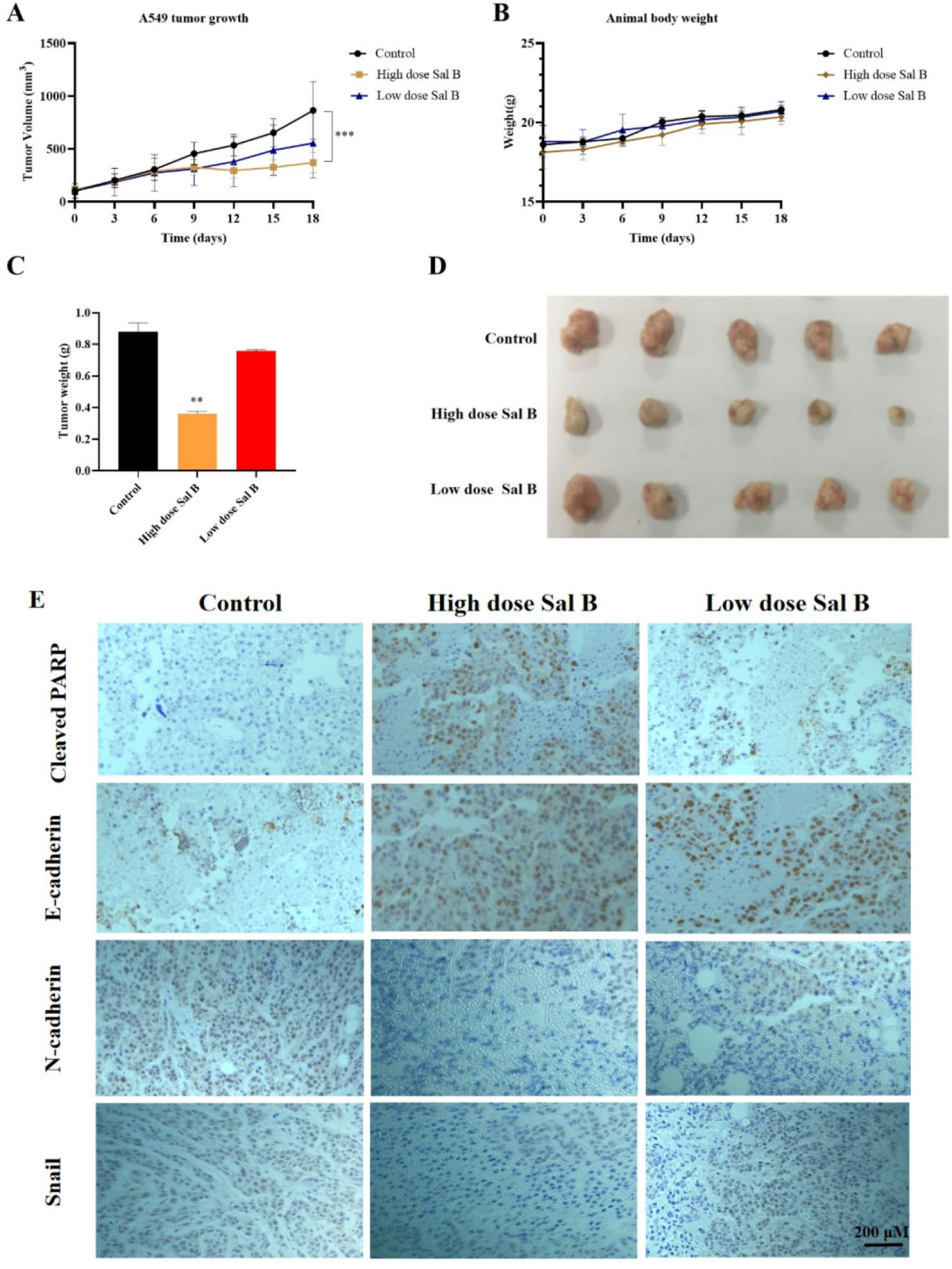
Antitumor efficacy of Sal B in A549 tumor-bearing mice. (A) Growth curve of implanted A549 xenograft in nude mice. (B) The body weight of the treatment with Sal B in the A549 mice model. (C) Tumor weight. (D) The resulting tumors were excised from the animals after treatment. (E) Immunohistochemistry of tumor sections. Scale bar, 200 µM. Data were expressed as mean ± SD, *n* = 6. **P < 0.01 and *P < 0.05 compared with the control group.

To investigate the in *vivo* antitumor activity of Sal B, an A549 xenograft mouse model was established. When the tumor volume reached 90–100 mm³, mice were treated for 18 days with 0.9% saline as control, low-dose Sal B (50 mg/kg), or high-dose Sal B (100 mg/kg). As illustrated in Figs. 5A-D, Sal B exhibited a significantly inhibitory effect on A549 tumor growth in comparison with the control group, with inhibition rates recorded at 64.8% for the high-dose group and 34.2% for the low-dose group, thereby substantiating a dose-dependent antitumor effect in *vivo*. It is noteworthy that Sal B treatment at both doses did not lead to significant weight loss, indicating a favorable safety profile. Furthermore, immunohistochemical analysis of tumor sections revealed markedly increased expression of cleaved PARP, an apoptotic marker, and E-cadherin, an epithelial marker, in Sal B-treated tumors. Conversely, the expression of mesenchymal markers N-cadherin and Snail was significantly reduced following Sal B treatment (Fig. 5E). Conversely, the expression of mesenchymal markers N-cadherin and Snail was significantly reduced in a dose-dependent manner following Sal B treatment (Fig. 5E). The findings indicate that Sal B induces apoptosis and inhibits metastasis within the tumor tissue.

### Sal B inhibits tumor growth on A549 tumor xenograft involved in the miR-23a/PTEN signaling pathway in *vivo*

**Fig. 6:**
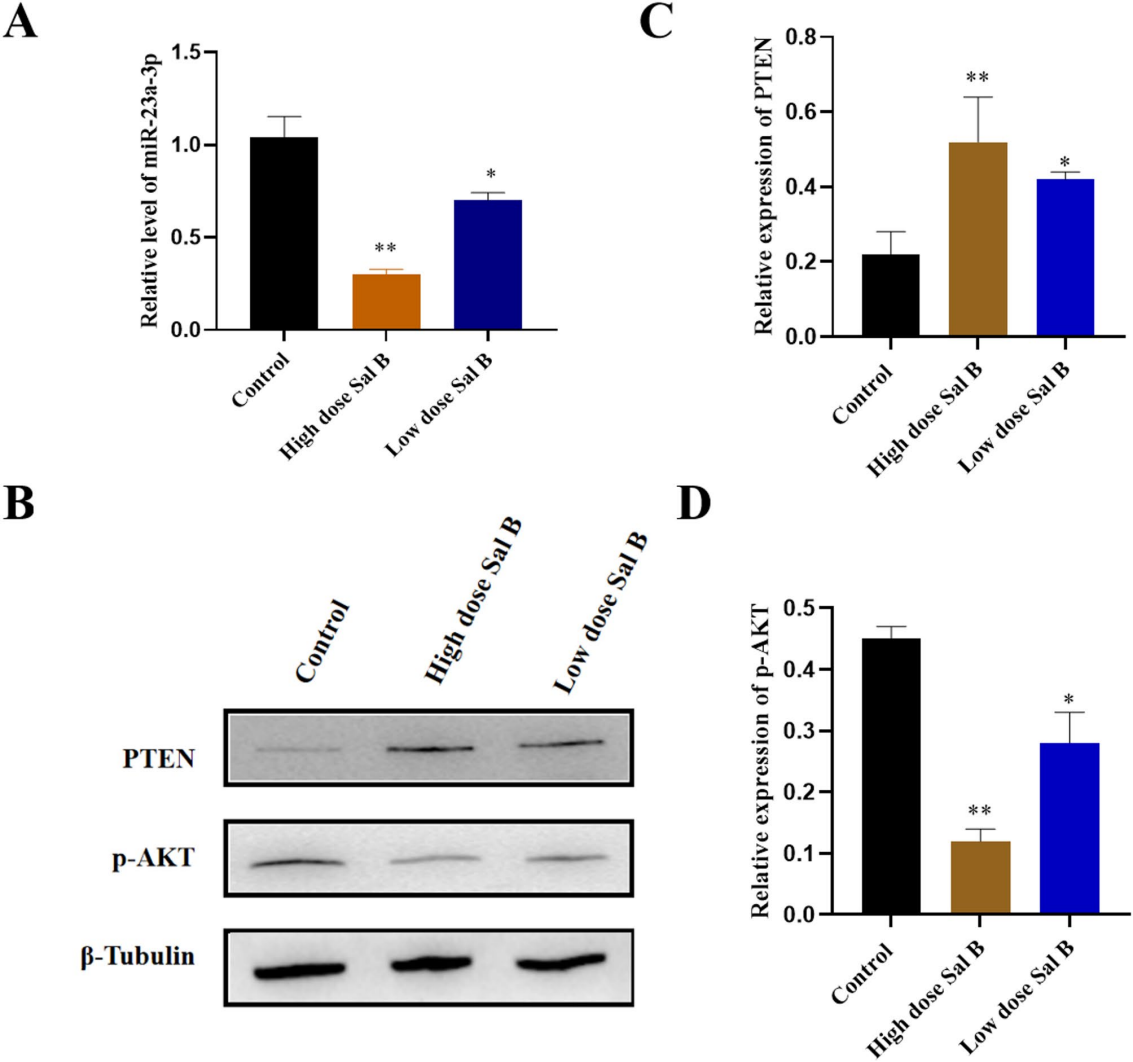
Effect of Sal B on miR-23a/PTEN signaling pathway in *vivo*. (A) RT-PCR analysis of miR-23a in the tumor tissues of mice treated with Sal B. (B) Western blot analysis of PTEN and p-AKT in the tumor tissues of mice treated with Sal B. The grouping of gels/blots were cropped from different parts of the same gels. (C) The protein levels PTEN was quantified by ImageJ and normalized to the β-tubulin protein content. (D) The protein levels p-AKT was quantified by ImageJ and normalized to the β-tubulin protein content. The bar graphs present the mean ± SD. Original blots/gels are presented in Supplementary Figure S5. Statistical analysis was performed by one-way ANOVA. **P < 0.01 and *P < 0.05 vs. control group.

To elucidate the mechanism by which Salvianolic acid B (Sal B) exerts its anti-tumor effect in A549 xenograft mouse models, RT-PCR and immunohistochemical analyses were conducted on tumor tissue samples. Initially, we quantified the expression levels of miR-23a in A549 mouse tumor tissues. As shown in Fig. 6, the RT-PCR analysis revealed that Sal B treatment resulted in a significant, dose-dependent reduction in miR-23a levels. Furthermore, Western blot analysis results indicated that Sal B treatment resulted in a significant upregulation of PTEN expression, whilst concurrently decreasing p-AKT levels. Collectively, these findings suggest that Sal B exerts its antitumor effects by modulating the miR-23a/PTEN/AKT signaling pathway.

### Discussion

NSCLC is the most common pathological type of lung cancer, with adenocarcinoma being the predominant subtype. Current clinical treatments, such as immunotherapy and targeted therapy, often cause toxic side effects that adversely affect patient prognosis^18^. Excitingly, phytochemicals derived from traditional Chinese medicine (TCM) have shown promising antitumor activity and can be applied in clinical treatment with fewer toxic side effects^19^. The results from the present study demonstrated that Sal B, a polyphenolic compound extracted from *Salvia miltiorrhiza*, exhibited in *vitro* and in *vivo* anticancer activity against NSCLC, which was reflected in the inhibition of A549 cell proliferation, migration, invasion, and EMT, the downregulation of miR-23a-3p and metastasis-related inhibition of PTEN/PI3K/AKT signaling.

Gefitinib, a selective epidermal growth factor receptor tyrosine kinase (EGFR TK) inhibitor, remains the classical drug used in the treatment of NSCLC^20^. Evidence from previous xenograft studies indicated that at the maximum tolerated dose of gefitinib (150 mg/kg), a 70.0% growth inhibition was observed in A549 tumors after two weeks^21^. In this study, Sal B (100 mg/kg) at a lower dose significantly suppressed tumor growth in the A549 xenograft model, achieving a 64.8% tumor-growth inhibition rate, which was comparable to that of gefitinib (150 mg/kg) in terms of the tumor inhibition rate (Fig. 5). It is noteworthy that the high dose of gefitinib (>100 mg/kg) caused skin toxicity in mice, and the body weight loss were significantly^22^. In contrast, Sal B is generally regarded as a safe natural compound. Sal B is widely administered by intravenous injection (i.p.) in clinic due to its low oral bioavailability (≈ 2.3%)^23^. Currently, available data support the safety of Sal B in therapeutics. He et al. (2023) reported that doses up to 300 mg/kg caused no toxicity in pregnant rats, and 100 mg/kg did not impair embryofetal development^24^. Based on this evidence, we selected intraperitoneal administration of Sal B (50 and 100 mg/kg) in this study to ensure adequate systemic exposure. Our results showed that Sal B significantly inhibited A549 xenograft growth in a dose-dependent manner without inducing significant body weight loss, suggesting its favorable therapeutic potential.

MicroRNAs (miRNAs) are critical regulators of gene expression, primarily at the post-transcriptional level. Accumulating evidence indicates that abnormal expression of miRNAs in the tumor microenvironment is involved in tumor progression^25,26^. The dysregulation of miRNAs in the tumor microenvironment was attributed to the alternation of oxidative stress^27^. Our previous work has demonstrated that Sal B could significantly reduce intracellular ROS levels in A549 cells to modulate oxidative stress and increased expression level of the tumor suppressor PTEN^19^. Therefore, we used computer-aided prediction tools, including miRDB, and identified the top 10 miRNAs that potentially target PTEN. Finally, we confirmed that miR-23a-3p was the most significantly downregulated miRNA in Sal B-treated A549 cells (Fig. 3). It has validated that miR-23a-3p could directly bind to the 3′-UTR of PTEN mRNA through dual-luciferase reporter assays^28^. Our transfection experiments further confirmed that over-expression of miR-23a-3p effectively reversed the increased protein levels of PTEN after Sal B treatment (Fig. 4). Taken together, these results suggest that the Sal B-induced reduction in ROS levels may account for the decreased expression of miR-23a-3p, which in turn contributes to the upregulation of PTEN.

It is well known that PTEN attenuates PI3K downstream signaling and negatively regulates EMT by antagonizing the PI3K/AKT pathway^29^. EMT is the basis of tumor metastasis and an important reason for tumor cells to gain metastatic potential. Our results showed that Sal B treatment suppressed the EMT process, as confirmed by upregulation of the epithelial marker E-cadherin and downregulation of mesenchymal markers, including N-cadherin and Snail. Notably, the changes in Sal B-regulated EMT progression may be attributed to the upregulation of PTEN expression and the reduction of the p-AKT levels caused by Sal B treatment (Figs. 2 and 6). Previous studies using the A549 cell line overexpressing wild-type or mutant PTEN, or employing PTEN siRNA, have demonstrated that PTEN plays a vital role in suppressing cell proliferation by inducing cell cycle arrest and apoptosis^30^. Therefore, it could be concluded PTEN knockout or knockdown may affect drug efficacy. Future studies will include PTEN knockdown or overexpression following Sal B treatment to further strengthen its action mechanism.

MAPK signaling is known to regulate EMT by modulating Smad2/3 phosphorylation, while PTEN/PI3K/AKT can also phosphorylate Smad3, thereby influencing EMT and cell proliferation^31,32^. In addition, Sal B has also been reported to inhibit the MAPK and Smad2/3 pathways in NSCLC cells, which is not unexpected given that natural products often exert multitarget effects^33^. Consequently, further research utilizing NSCLC models both in *vivo* and in *vitro* is necessary to investigate the two potential interaction mechanisms of Sal B.

In summary, our results demonstrate that Sal B can downregulate the expression of miR-23a-3p, leading to an increase in PTEN expression, which subsequently inhibits the PI3K/AKT pathway and contributes to the suppression of EMT in NSCLC. However, a significant issue that must be addressed before Sal B can be utilized in the treatment of NSCLC is its limited oral bioavailability. Future research efforts will focus on identifying alternative formulations, such as lyophilized powders, dry-powder inhalers, and lipid-based carrier formulations. Additionally, it was primarily conducted in A549 cells, which represent a single NSCLC subtype. Given the genetic and molecular heterogeneity of NSCLC, including the variability of PTEN/PI3K signaling status, further validation across diverse NSCLC cell lines and patient-derived xenograft models is warranted.

## Supplementary Information

Below is the link to the electronic supplementary material.

Supplementary Material 1

## Data Availability

The datasets used and/or analyzed during the current study are available from the corresponding author upon reasonable request.

## Declarations

### Arrive statement

This study was conducted in accordance with the ARRIVE guidelines. All methods were conducted in accordance with the relevant guidelines and regulations.
