# Supplementary material for: Salvianolic acid B inhibits the proliferation and metastasis of A549 lung cancer cells via miR-23a/PTEN/AKT pathway
Source: Sci Rep. 2025 Dec 22;15:45066. doi: 10.1038/s41598-025-32336-9 (PMC12748603; doi:10.1038/s41598-025-32336-9)
Supplement: Supplementary file 1 — Supplementary Material 1 [file 41598_2025_32336_MOESM1_ESM.pdf]

Supplementary information

Salvianolic acid B inhibits the proliferation and metastasis of A549 lung cancer cells via miR-23a/PTEN/AKT pathway

Ye Yang[1], Lei Huang[1*], Li Dai[2], Xin Zhou[3], Bingjun Qian [3*]

Supplementary Figure 1. (Fig. S1) Overexpressed miR-23a-3p in NSCLC cell lines A549 was confirmed using RT-PCR after transfection.

Supplementary Figure 2. (Fig. S2) Effect of Sal B on A549 tumor metastasis and the PTEN signaling pathway in *vitro*.

Supplementary Figure 3. (Fig. S3) Sal B suppressed the proliferation and metastasis of A549 cells by downregulating miR-26a-3p

Supplementary Figure 4. (Fig. S4) Effect of Sal B on PTEN protein.

Supplementary Figure 5. (Fig. S5) Effect of Sal B on miR-23a/PTEN signaling pathway in *vivo*.

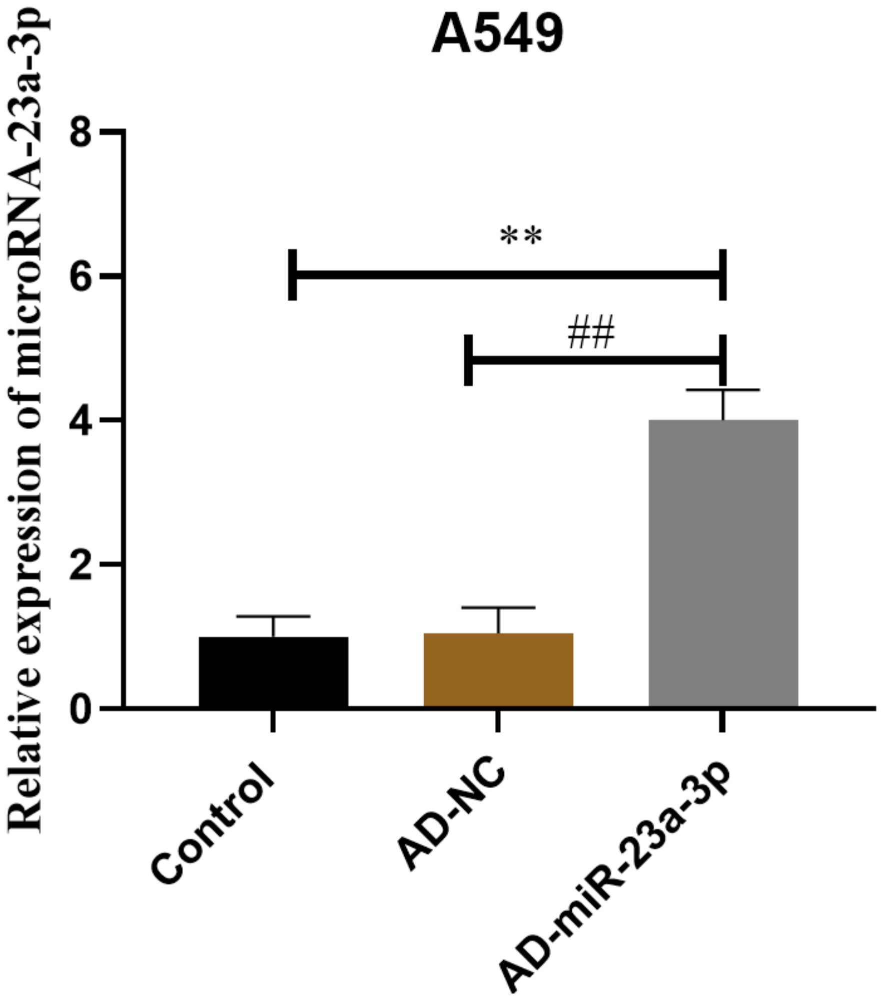

Fig. S1 Overexpressed miR-23a-3p in NSCLC cell lines A549 was confirmed using RT-PCR after transfection. Data were presented as the mean ± standard deviation of three independent experiments. **P<0.01 vs control. ##P<0.01 vs AD-NC. AD-NC: adenoviruses harboring mimic non-specific control; Control, negative control.

Fig. 2A

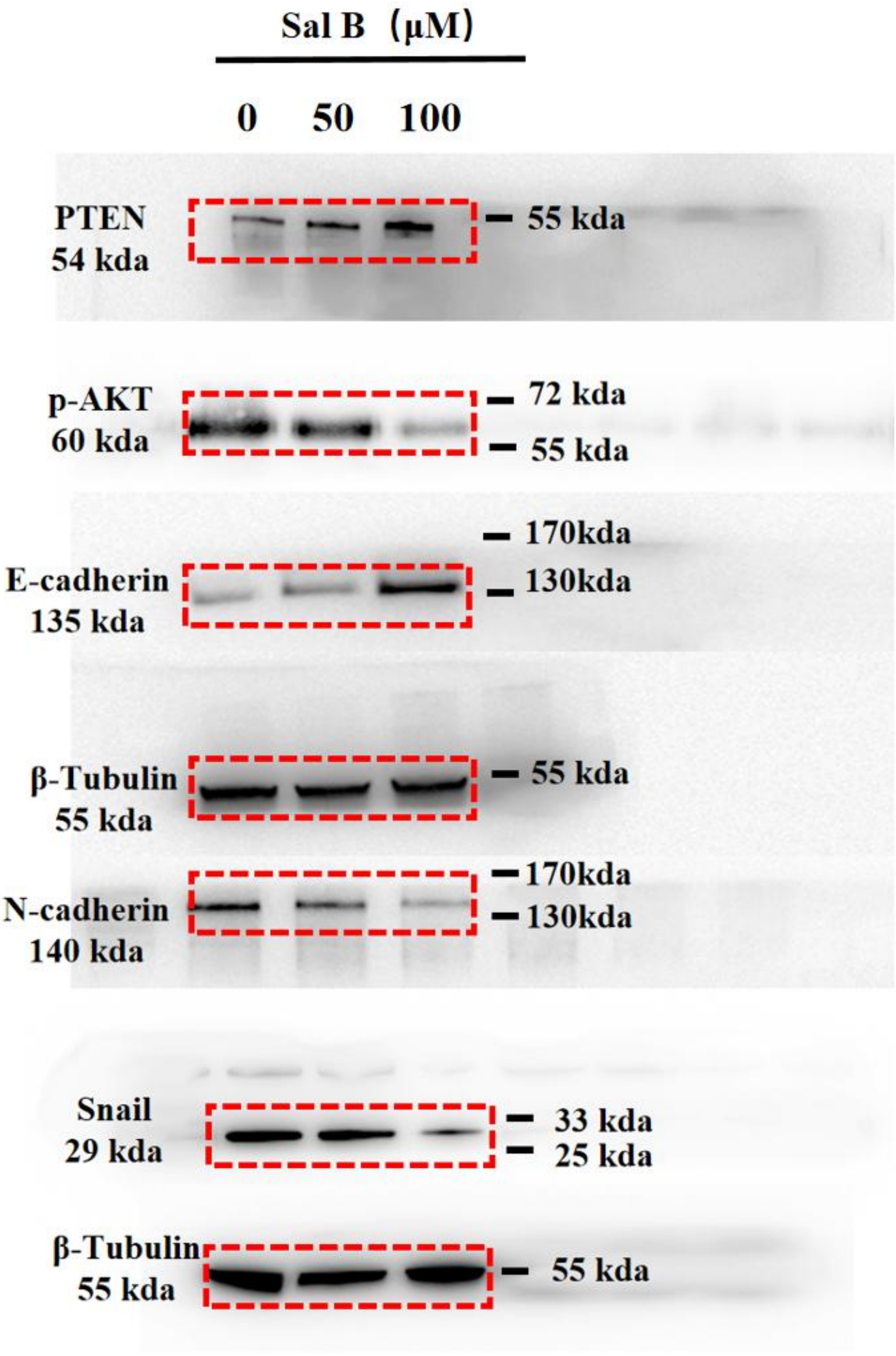

Figure S2 Uncropped blots of all western blot results.

Fig. 3E

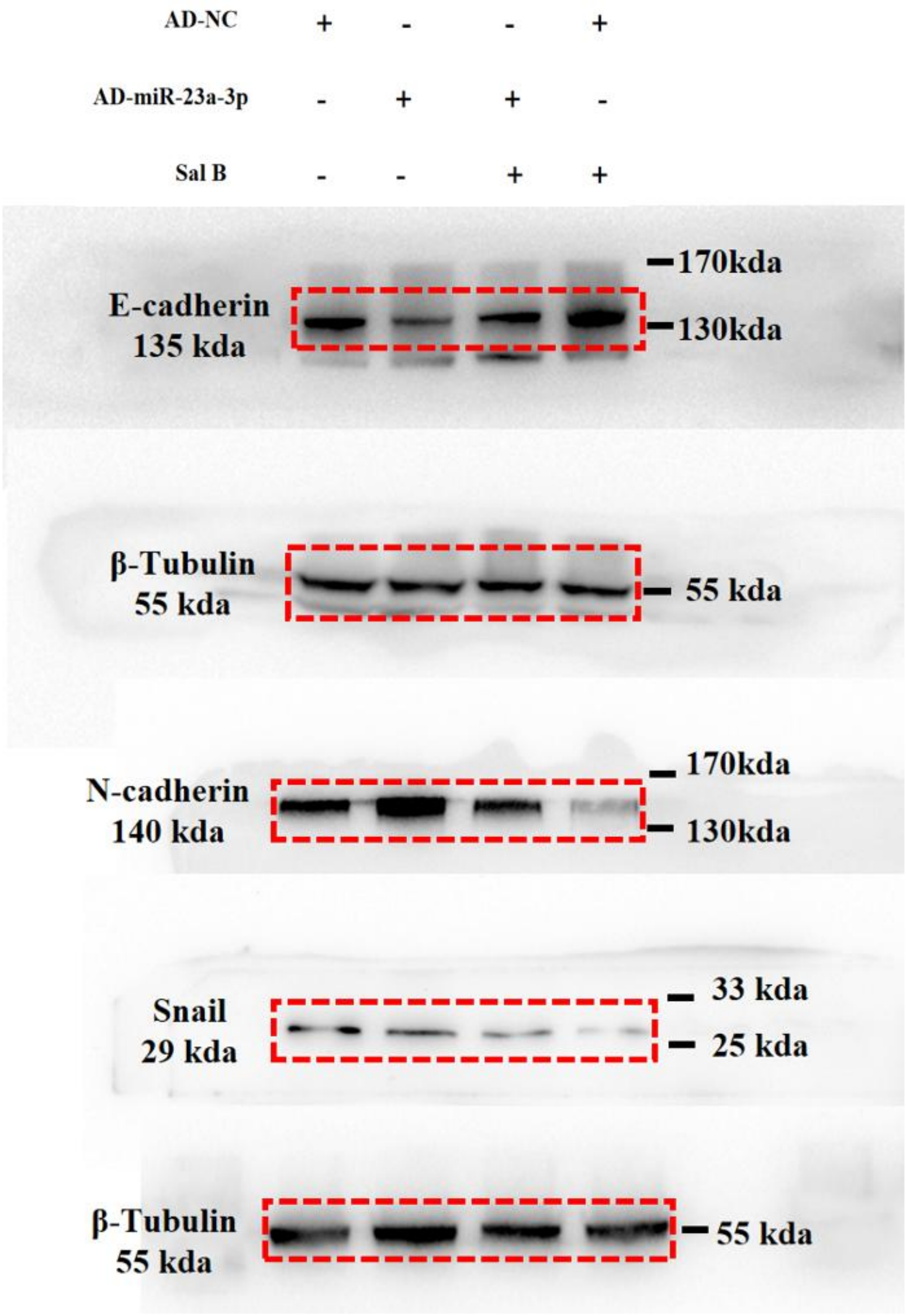

Fig. S3 Uncropped blots of all western blot results.

Fig. 4A

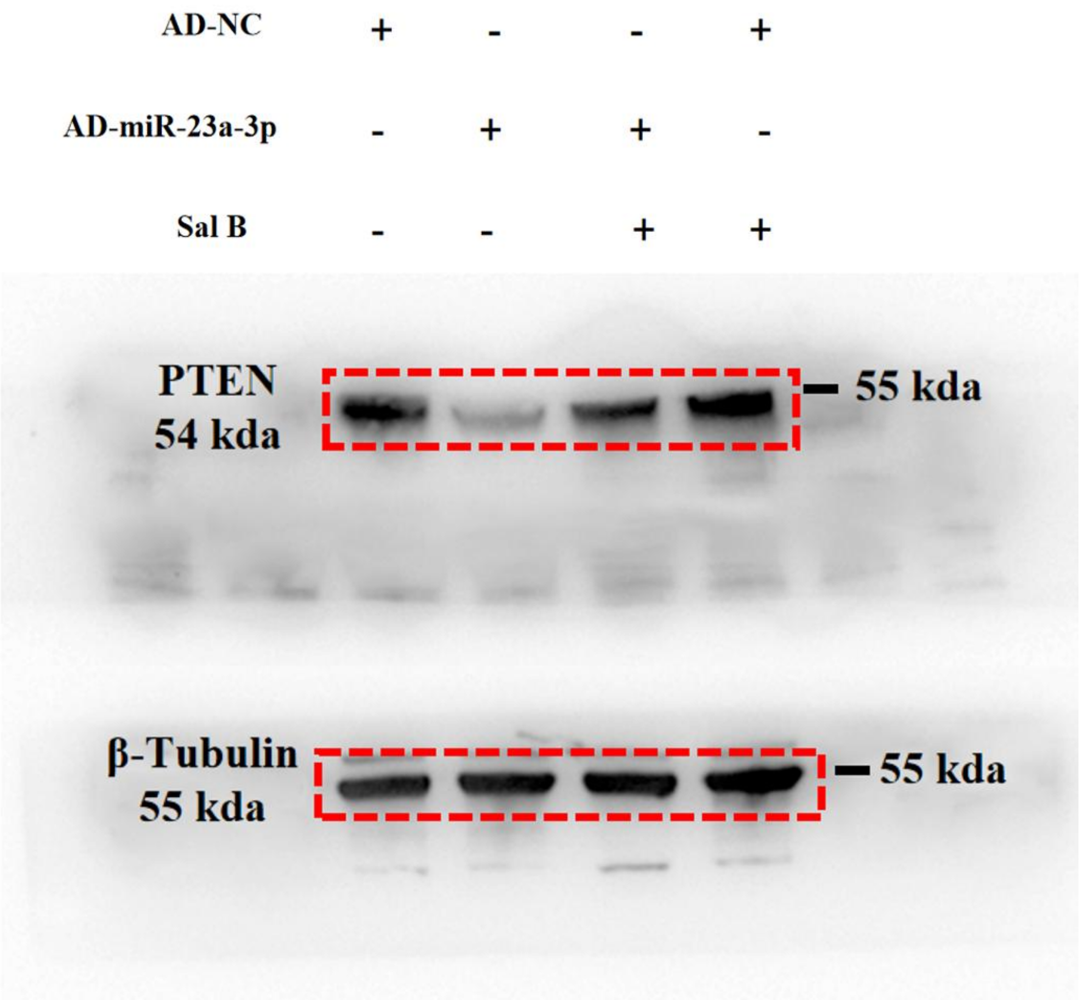

Fig. S4 Uncropped blots of all western blot results.

Fig. 6B

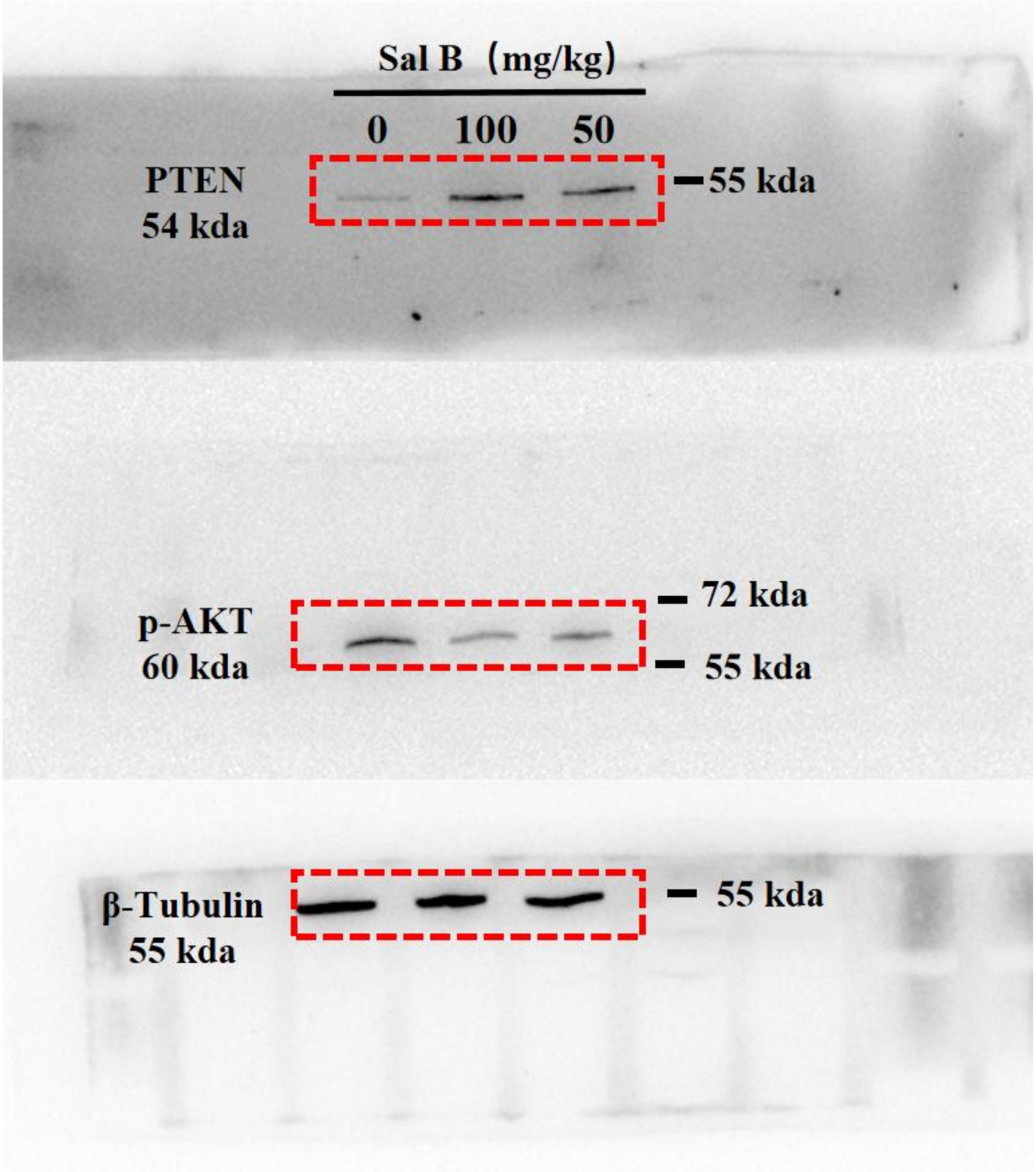

Fig. S5 Uncropped blots of all western blot results.
